# Epidemiology of neurodegenerative diseases in the East African region: A meta-analysis

**DOI:** 10.3389/fneur.2022.1024004

**Published:** 2022-11-17

**Authors:** Hope Onohuean, Abraham Olutumininu Akiyode, Oluwole Akiyode, Sharon Iyobor Igbinoba, Abdullateef Isiaka Alagbonsi

**Affiliations:** ^1^Biopharmaceutics Unit, Department of Pharmacology and Toxicology, Kampala International University Western Campus, Ishaka, Uganda; ^2^Biomolecules, Metagenomics, Endocrine and Tropical Disease Research Group (BMETDREG), Kampala International University Western Campus, Ishaka, Uganda; ^3^Department of Biology, College of Arts and Sciences, University of Texas of the Permian Odessa, TX, United States; ^4^Biological and Environmental Sciences Department, Kampala International University, Kampala, Uganda; ^5^Department of Clinical Pharmacy and Pharmacy Administration, Faculty of Pharmacy, Obafemi Awolowo University, Ile-Ife, Osun State, Nigeria; ^6^Department of Clinical Biology (Physiology Unit), School of Medicine and Pharmacy, College of Medicine and Health Sciences, University of Rwanda, Huye, Rwanda

**Keywords:** epidemiology, potentials, neurodegenerative diseases, East Africa, meta-analysis

## Abstract

**Introduction:**

There is a scarcity of epidemiological data on neurodegenerative diseases (NDs) in East Africa. This meta-analysis provides the regional prevalence of NDs, their contributing factors, and evidence of change over time concerning gender per age or year.

**Methods:**

Articles were retrieved from electronic databases following the PRISMA standard.

**Results:**

Forty-two studies were reviewed, and 25 were meta-analyzed with a random-effects model. The pool estimate proportion of 15.27%, 95% CI (0.09–0.23) (I^2^ = 98.25%), (Q = 1,369.15, *p* < 0.0001) among a population of 15,813 male/female and 1,257 with NDs. Epidemiological characteristics associated with NDs include Dyskinesias prevalence 55.4%, 95% CI (13.5; 90.9), I^2^ (96%) and subsistence farming prevalence 11.3%, 95% CI (5.8; 20.9), I^2^ (99%). Publication bias by Egger test was (z = 4.1913, *p* < 0.0001), while rank correlation test using Kendall's model was (tau = 0.1237, *p* = 0.3873). Heterogeneity (R^2^ design = 5.23%, *p* design < 0.0001; R^2^ size = 52.163%, *p* size < 0.001; and R^2^ period = 48.13, *p* period < 0.0001. Covariates (R^2^ design + size + period = 48.41%, *p* < 0.001).

**Conclusion:**

There is a high prevalence of NDs in the East African region, which could impact life expectancy, morbidity, and quality of life. Thus, early screening and regular surveillance could assist in management strategies.

## Introduction

The loss of function in the brain's nerve cells and the peripheral nervous system causes neurodegenerative diseases (NDs). Alzheimer's disease (AD), Parkinson's disease (PD), Huntington's disease (HD), and Lou Gehrig or amyotrophic lateral sclerosis (ALS) are the major neurodegenerative disorders, which are of public health concerns (1–4) and characterized by the inevitable degeneration of specific neuronal clusters (5). This group of illness provides a significant clinical challenge due to its progressive nature (6, 7), in which nerve cells in the brain and peripheral nervous system lose their ability to function over time and eventually die and can only be delayed, not completely stopped, once it has started.

In most surveys around the world, annual incidence rates of NDs are estimated to be 10–15 per 100,000, 2% of which are people over the age of 65 years (8). According to a 2019 estimate, ~50 million individuals worldwide suffered from NDs that resulted in dementia, and this number is expected to rise to 152 million by 2060 (9–11).

In the United States (US), the most prevalent and burdensome neurological illnesses are tension-type headache (TTH) [121.6 (95% UI, 110–133) million people], migraine [68.5 (95% UI, 64–73) million people], stroke [7.8 (95% UI, 7.4–8.2) million people], AD and other dementias [2.9 (95% UI, 2.6–3.2) million people], and spinal cord injuries (SCI) [2.2 (95% UI, 2.0–2.3) million people] (12).

In sub-Saharan Africa, population-based prevalence of PD, ALS, and HD range from 10 to 235 per 100,000, 5 to 15 per 100,000, and 3.5 per 100,000, respectively, while their corresponding hospital-based prevalence are 0.41 to 7.2%, 0.2–8.0 per 1,000, and 0.2 to 46.0 per 100,000, respectively (13). Nevertheless, data on the prevalence and epidemiological potentials of neurological illnesses in the communities are sparse in Sub-Saharan Africa, especially those living in hard-to-reach settlements. In most rural areas, residents often suffer the most due to a lack of knowledge about the symptoms and adequate early diagnosis or medical examinations for these neurological diseases and are left out of the epidemiological survey.

East Africa is a region consisting of many low-income communities that have reported several cases of malaria (14, 15), HIV/AIDs (16, 17), and diarrhea (18–21) infections as well as sporadic multi-drug resistance (22, 23). Various forms of NDs have also been reported to be prevalent in the region. In the Mukono district of Uganda, for instance, the frequencies of peripheral neuropathy (46.2%), chronic headaches (26.4%), epilepsy (8.5%), pain syndromes (7.5%), stroke (6.6%), and tremors/Parkinson disease (3.8%) were reported to be high. Moreover, the crude prevalence rates (95% CI) of stroke, epilepsy, and peripheral neuropathy were reported as 14.3% (8.5–24.1), 13.3% (7.7–22.8), and 33.7% (23.9–47.4), respectively (24). In the rural Hai district of Tanzania, the age-adjusted prevalence per 1,000 of the most common neurological disorders was tremor (48.2), headache (41.8), stroke (23.0), peripheral polyneuropathy (18.6), upper limb mononeuropathy (6.5), and parkinsonism (5.9) (25).

Exploring the epidemiology and factors contributing to the prevalence of NDs is critical for identifying patterns of disease predisposition, environmental clustering, and medication responses mostly at regional, communal, and individual levels (23, 26). The knowledge of whether NDs incidence and/or prevalence is changing would have substantial scientific and therapeutic implications, aside from the need of having accurate, up-to-date numbers for developing services to meet the needs of the population suffering from them.

There is scarce information on the prevalence of neurological disorders and epidemiological potentials in populations of other East African regions to identify research opportunities and suggest preventative or mitigation solutions. Again, there is a lack of presentation of published East African epidemiologically relevant studies. Here, we provide the first epidemiological assessment of major neurological illnesses in the East African region, giving information on the prevalence of such disorders. Thus, a decrease would imply that future rates are partially adjustable and effectively addressing the changeable risk factors could delay, if not completely prevent the illness. The specific objective includes (1) providing regional estimates of NDs prevalence and incidence, (2) investigating factors that contribute to estimating heterogeneity (study location {Countries}, NDs types, study design, sample size, study period), and (3) finding evidence of change in NDs prevalence and/or incidence over time concerning gender per age or year. This research study updates and expands the prior findings on the epidemiology of this condition.

## Materials and methods

The statement (Appendix 1 Table) of the Preferred Reporting Items for Systematic Reviews and Meta-Analysis (PRISMA) (23, 27) was used in this study. The Boolean keywords in the form of title words or medical subject headings were conducted as a literature search using the electronic databases: PubMed, Web of Science (WOS), SCOPUS span 1980 and November 2021. The keywords, neuro disease or neurodegenerative disease, detail search algorithms, or key terms in the additional information (Appendix 2 Text) were used to assess the prevalence, epidemiology, population, and survey data within the East African region. The datasets were combined on RStudio versions 4.0.5 using the bibliometrix R package (28), while the removal of duplicates and normalization of variables were done using ScientoPy and fBasics R-packages (29), followed by a hand check of the reference lists of all retrieved studies to add any relevant articles. Two reviewers (HO and AOA) did the literature search independently.

### Selection criteria

Studies that matched the following criteria were included, such as (1) data on the population-based prevalence of NDs or epidemiological and observational studies on NDs reported in East Africa, (2) NDs diagnosis made for both males/females across all ages; (3) Prognosis of NDs based on the opinions of a qualified medical practitioner, electrophysiological test, or medical records indicating a diagnosis of NDs according to the International Classification of Diseases and Codes; (4) full-text articles written in English that are available; (5) Diagnostic and Statistical Manual of Mental Disorders (DSM-IV); (6) Identification and Interventions for Dementia in Elderly Africans (IDEA) screen; (7) International HIV Dementia Scale (IHDS); (8) Montreal Cognitive Assessment test (MoCA); (9) Based on the modified McDonald's criteria.

The following studies were excluded: (1) conference abstracts' documents or meeting abstracts, letters, or reviews; full texts without raw data, total population, positive cases, and duplicate publications; (2) NDs were not based on objective examination or medical records and involved self-reported cases; (3) questionnaires or app technology-based studies; (4) Dementia-associated with the treatment of HIV and other terminal diseases; (4) articles published in languages other than English.

Based on these criteria, two reviewers (H. O. and A. O. A.) independently selected the studies for final inclusion while a third author (S. I.) doubled-check, arbitrated, and resolved disagreements between the two reviewers.

### Data extraction and outcomes of interest

Following the inclusion and exclusion criteria, information on the first author names, publication year, the total population, number of positive cases, country of study, study source, study period, and study type were identified and extracted from results, discussions, figures and tables in the qualified articles as the meta-analysis indices by two investigators independently (HO and AOA) and double-checked by a third investigator (S. I). Afterward, documentation of homogeneity or consistency and heterogeneity across studied populations was done and further statistical analysis was based on requirements for the study as conceptualized by the investigators.

### Assessment of data quality and statistical analysis

The Newcastle–Ottawa Scale (NOS), proposed by the Agency for Healthcare Research and Quality (AHRQ), was used to assess methodological quality found in (http://www.ohri.ca/programs/clinicalepidemiology/oxford.asp). This rating employs a star system to rate the quality of a study in three areas: study group selection, group comparability, and outcome measurement.

The raw proportions were used to compute the regional population-based NDs prevalence from 27 studies, and the Wilson method was employed to calculate 95% confidence intervals (CIs). The study's random-effects meta-analyses weighting was done by estimating the summary effect size (weighted average proportion) to calculate the pooled effect size based on the individual effect sizes and their sampling variances *via* the argument method = “DL” (using the restricted maximum-likelihood estimator). To improve the statistical properties, the logit transformation was conducted to get the pooled prevalence since we have a mean proportion of 0.03 across the studies (30). The effects of examined homogeneity or consistency and heterogeneity across studied populations were measured using sensitivity and influence meta-regression analysis of sample size and period. A Forest plot for the overall and the mixed effects model for the between-study variation of subgroups analysis (design, East Africa region (location) as well gender per year/per age distribution were generated. The Funnel plots for comparison of publication bias were conducted according to asymmetry Egger's test for this purpose and further examined for the significance of the bias using the rank correlation test and Kendall's model. All analyses were two-tailed with *p* < 0.05 level of significance and were conducted in the statistical software R 4.0.5. packages (31, 32).

## Results

### Literature search summary

#### Summary of included studies

**Figure 1:**
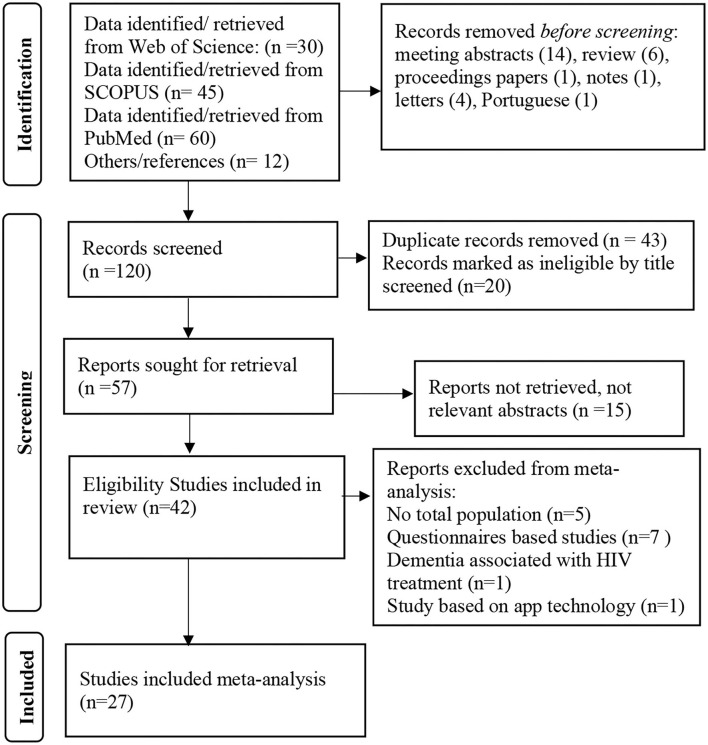
Study selection flowchart.

Our search found a total of 147 NDs articles from the three databases reported in the East African region between 1980 and 2021. After removing duplicates and studies that were not relevant, the abstracts of the remaining studies were read, and 42 articles from different countries in the East African region with possibly relevant studies were reviewed, the details of which are in the flowchart (Figure 1). Thirty-one studies were from Tanzania, five were from Uganda, one from Ethiopia, and five were from Kenya, and they can be found in Supplementary Figure 1. The sample sizes of the studies ranged from 21 to 161,000 males and females in the East Africa regional NDs prevalence estimate. The retrieved age ranged from 32 years to 100 years old at the time of diagnosis, and 42 articles (Table 1) were systematically reviewed while 25 published studies were included in the meta-analysis. Based on the systematic review, a total population of 26,762 were tested for the NDs and a prevalence of a total of 1,629 people (6.09%): male 600 (36.83%) and female 995 (61.08%) were observed. While the gender of 34 positive cases was not identified in the included studies.

**Table 1:** Overall characteristics of the included studies.

**First authors**	**Nation/region**	**Study type**	**Age**	**Degenerative disease**	**Study period**	**Sample size**	**Score**
**Community-based prevalence study**							
Kioy (33)	Kenya	Retrospective study	17–39 y	Multiple Sclerosis	10 years	2,831	7
Jamal (34)	Kenya	Cohort/ Retrospective Descriptive study	NA	Multiple Sclerosis	14 months	1,237	7
Dotchin (35)	Tanzania	Community-based prevalence study	>70 y	Dementia	NA	126	6
Dotchin (35)	Tanzania	Community-based prevalence study	>70 y	Parkinson's	NA	126	6
Scrimgeour (36)	Tanzania	Community-based prevalence study	25–>80 y	Huntington's	NA	NA	5
Aris (37)	Tanzania	Community-based prevalence study	>70 y	Parkinson's	5 years	29	7
Winkler (38)	Tanzania	Community-based prevalence study	50–110 y	Parkinson's	5 months	1,569	6
Miller (39)	Tanzania	Community-based prevalence study	NA	Parkinson's	NA	NA	4
Longdon (40)	Tanzania	Cross sectional Community Based-study	>70 y	Dementia	NA	1,198	6
Paddick (41)	Tanzania	Cross sectional Community Based-study	>60 y	Dementia	12 months	3,011	7
**Cross-sectional population-based study**							
Mubangizi (42)	Uganda	Cross sectional population based-study	>60 y	Dementia	1 month	400	6
**Cross-sectional qualitative study**							
Mushi (43)	Tanzania	Cross sectional qualitative study	>70y	Dementia	3 months	41	7
Kaddumukasa (24)	Uganda	Cross sectional Study	26–47 y	Parkinson's	6 months	98	7
Kankongi (44)	Uganda	Descriptive cross-sectional study	>60 y	Alzheimer's'	1 month	30	7
**Hospital-based study**							
Paddick (45)	Tanzania	Hospital based study	>65 y	Dementia	3 months	205	7
Winkler (38)	Tanzania	Prevalence Hospital based study	>32 y	Parkinson's	8 months	740	7
Sacktor (46)	Uganda	Prevalence Hospital based study	>18 y	Dementia	5 months	117	7
Dotchin (47)	Ethiopia	Hospital-based study	54.6 y (m)	Parkinson's	NA	NA	4
**Questionnaire**							
Chen (48)	Kenya	Cohort	>65 y	Alzheimer's'	NA	200	6
Dotchin (49)	Tanzania	Questionnaires	>35 y	Parkinson's	1 year	160,456	7
Mashana (50)	Tanzania	Qualitative research, semi structured interviews and focus group discussions	>35 y	Parkinson's	1 month	62	7
Sacktor (51)	Uganda	Qualitative study	NA	Dementia	16 months	60	7
Hindley (52)	Tanzania	Qualitative study	>70 y	Dementia	3 months	56	7
**Interventional study**							
Paddick (53)	Tanzania	Interventional study	>75 y	Alzheimer's'	NA	66	6
**Observational repeated measure design**							
Rochester (54)	Tanzania	Observational repeated measure design	45–100 y	Parkinson's	3 weeks	21	7
Paddick (55)	Tanzania	Follow up study	>70 y	Dementia	48 months	77	7
Screening based on app technology
Paddick (41)	Tanzania	Screening based on app technology	>60 y	Dementia	4 months	3,011	7
**Cohort**							
Fothergill (56)	Kenya	Ethnographic fieldwork	33–81 y	Parkinson's	10 months	NA	7
Kisoli (57)	Tanzania	Cohort	>70 (70–79) y	Parkinson's	10 months	2,232	7
Kisoli (57)	Tanzania	Cohort	>70 (70–79) y	Dementia	10 months	1,198	7
Dotchin (58)	Tanzania	cohort	37–94 y	Parkinson's	3 years	161,000	7
Kellet-Wright (59)	Tanzania	Cohort	>50 y	Dementia	4 months	820	7
Paddick (60)	Tanzania	Cohort	>70 y	Dementia	NA	296	6
Paddick (60)	Tanzania	Cohort	>70 y	Alzheimer's'	NA	296	6
Paddick (60)	Tanzania	Cohort	>70 y	Dementia	6 months	1,198	7
Paddick (60)	Tanzania	Cohort	>70 y	Alzheimer's'	6 months	1,198	7
Paddick (60)	Tanzania	Cohort	>70 y	V. Dementia	6 months	1198	7
Paddick (61)	Tanzania	Cohort	>65 y	Dementia	7 months	466	7
Paddick (62)	Tanzania	Cohort	>60 y	Dementia	NA	507	6
Masika (63)	Tanzania	Cohort	>60 y	Dementia	1 month	202	6
Kwasa (64)	Kenya	Cohort	39 y(m)	Dementia	1 month	30	7
Matuja (65)	Tanzania	Cohort	40–78 y	Parkinson's Disease	4 years	1,908	7

(m), mean age; NA, non available; V, Vascular; y, years.

#### Quality assessment

Supplementary Table 1 show the quality evaluation scores of the included studies, and Table 1 shows the details of the assessment questions in domains for each article. The NOS variables and comparability received no stars to any of the examined studies because comparative studies were not reported in the included publications. For the other studies, quality scores range from 4 to 7. Twenty-eight studies received 7 points, 11 studies received 6 points, a study received 5 points, and 2 studies received 4 points out of the maximum 8 points.

#### East Africa regional prevalence estimate of NDs

Upon removal of two outer liars in the analysis (35, 61), a total of 15,813 people, both male and female, were included in the 25 studies (33–35, 37, 38, 40–45, 48, 53–55, 57–62, 65, 66), and a prevalence of 7.95% (1,257) was recorded on the regional-based epidemiological potential of NDs. Also, the prevalence of NDs is apparently increasing in females per year and age in this regional meta-analysis, as depicted in Supplementary Figures 1, 2. The design distribution of these studies include cross-sectional study (*n* = 3), a community-based prevalence study (*n* = 5), hospital-based study (4), follow up study (1), while regional cohorts (*n* = 14) included data on the population-based prevalence of NDs in the studied region (Table 1).

**Figure 2:**
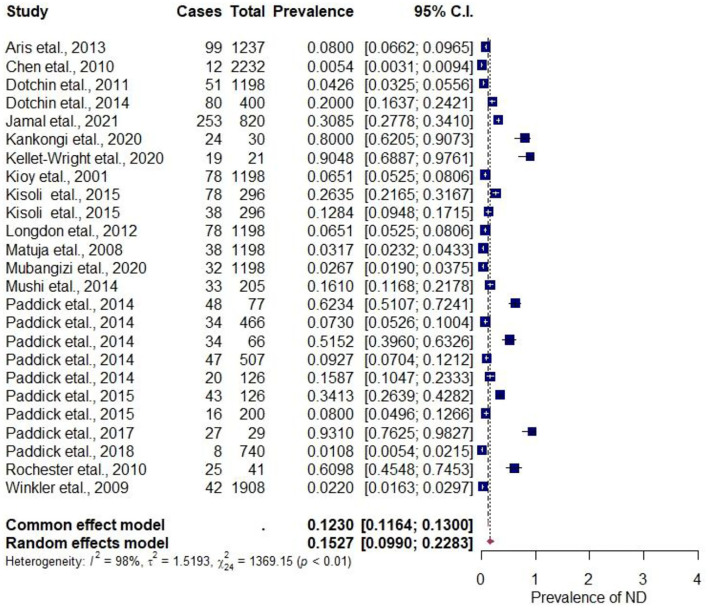
Forest plot for the prevalence of NDs in the regional-based studies.

The pooled estimate of ND prevalence calculated for the 25 east Africa regional base epidemiological potentials studies is as follows. The pool estimate proportion of 15.27%, 95%CI (0.09–0.23) with an (I^2^ = 98.25%) by random effect model, implicates a high proportion between studies. A significant Q statistic of (Q = 1369.15, *p* < 0.0001) was observed, indicating that the included studies do not share a common effect size. Therefore, overall, our NDs prevalence meta-analysis has substantial heterogeneity, as depicted in Figure 2. The evaluation of publication bias by a funnel plot (Supplementary Figure 4) using the Egger test model (*P* < 0.0001) indicates a significant level of publication bias.

The Funnel plots were used to determine the publication bias. Each point denotes a separate study on the designated association. The vertical line denotes the mean effect size. However, the points are dispersed asymmetrically, which shows publication bias. While the linear regression test of funnel plot asymmetry using the Egger test signifies a bias (z = 4.1913, *p* < 0.0001), indicating a significant publication bias (Supplementary Figure 4). On the other hand, the rank correlation test for funnel plot asymmetry using Kendall's model indicates tau = 0.1237 and *p* = 0.3873.

#### Source of heterogeneity analysis for the NDs prevalence estimate: Meta-regression

To examine the probable sources of heterogeneity observed in the visual forest plot and the baseline analysis of the included studies, five definite covariates were investigated. In univariate meta-regression analyses, the countries (Tanzania or Kenya or Uganda) and NDs types (Multiple Sclerosis or Parkinsons or Dementia or Alzheimers) were not significantly associated with the NDz prevalence (*p* = 0.166; R^2^ = 0.00%) (*p* = 0.8464; R^2^ = 0.00%). However, there was a significant estimate for the covariates analysis of the study design (Cohort or Other), sample size (less or more than 1,000), and study period (months or above 1 year). The R^2^ (amount of heterogeneity accounted for) and *p*-values for each covariate estimate are as follows: R^2^ design = 5.23%, *p* design < 0.0001; R^2^ size = 52.163%, *p* size < 0.001; and R^2^ period = 48.13, *p* period < 0.0001, respectively. Additionally, a subsequent multivariate mixed-effects meta-regression model was developed based on the study's (design, size, and period), with each of these variables showing significant associations with the pooled prevalence heterogeneity. These three covariates significantly accounted for 48.41% of the heterogeneity in the ND prevalence estimate (R^2^ design + size + period = 48.41%, *p* design + size + period < 0.001).

#### Variations in East Africa regional ND prevalence: Subgroup analysis

**Figure 3:**
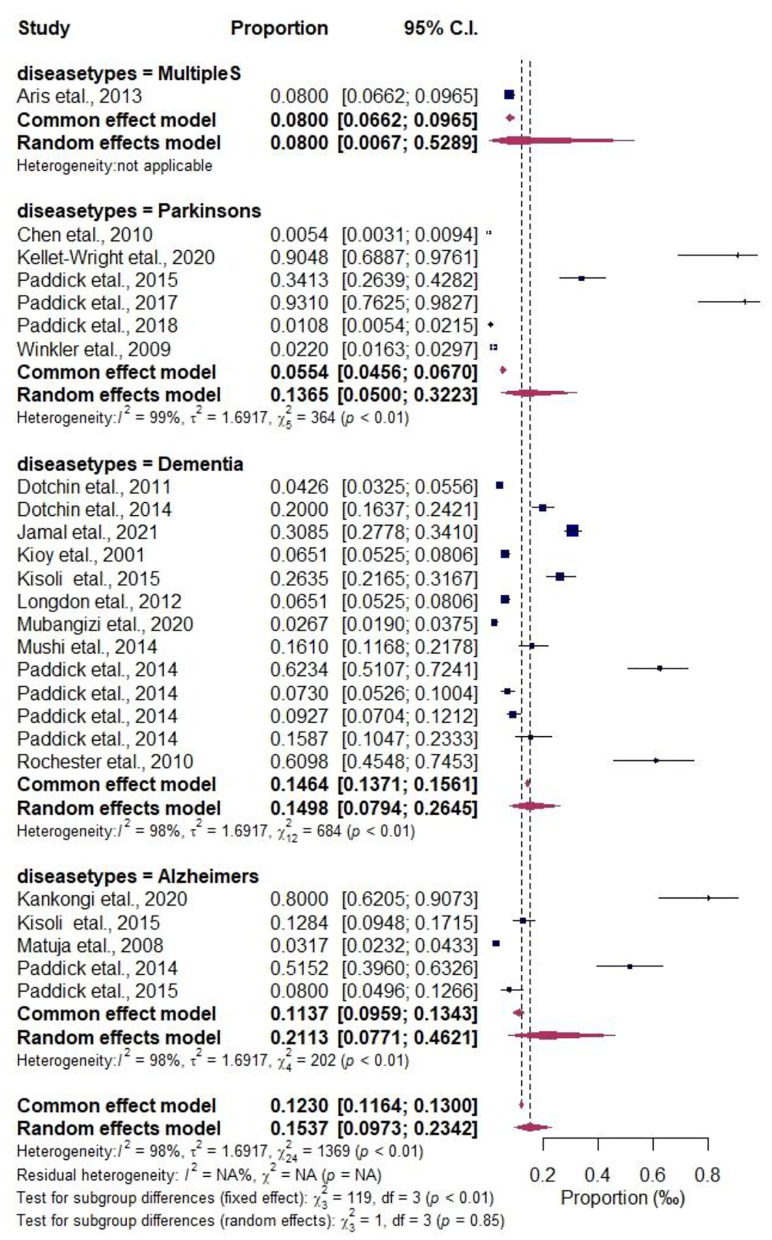
Forest plot of the subgroup analysis by NDs types.

The potential variations of study design (cohort or others) categorical variables, the others have the highest NDs prevalence estimate 44.94%, CI (0.272–0.640), I^2^ = 97.21%, to the cohort group 5.60%, CI (0.031–0.099), I^2^ = 98.49% in Supplementary Figure 3. Subgroup analysis by study size revealed the high NDs prevalence in < 1,000 size 27.97% CI (0.192–0.388), I^2^ = 96.58% to that of more than 1,000 sample size 3.46%, CI (0.022–0.054) as depicted in Supplementary Figure 4. The study period subgroup analysis reveals the most prevalence in years 22.87% CI (0.105–0.428), I^2^ = 96.15% to that of study period in months 12.12%, CI (0.069–0.204), I^2^ = 96.40% as shown in Supplementary Figure 5. For the individual subgroups, variations that were not significantly associated with the NDs prevalence [East Africa region (Kenya or Tanzania or Uganda) in Supplementary Figure 6 and NDs types (Multiple Sclerosis or Parkinsons or Dementia or Alzheimers)] are shown in Figure 3.

The Forest plots for the subgroup analysis of regional-based NDs prevalence including the subgroup analysis by research period, sample size, and East African countries are shown in Supplementary Figures 5–8. The prevalence of the subcategories according to two epidemiological characteristics including comorbidity and subsistence farming are also shown in Table 2. The comorbidities reported in the included articles by authors categorized as others include (chronic choreoathetosis, progressive dementia) Scrimgeour (36) (abnormalities of sympathetic and parasympathetic functions) Aris et al. (37) (visual disturbances and somatic sensorimotor disturbances) Kioy et al. (33) account for 93.1%, and dyskinesias, spinal cord pathology, HIV, cognitive impairment 55.4, 34.1, 28.2, and 20.1%, respectively. On the other hand, the economic status was categorized into two: subsistence farmers and others such as (workers, retired, student, unemployed, employed, house wife/husband) as reported in the included articles. Subsistence farming accounts for 11.3% prevalence, while others account for 5.7%.

**Table 2:** Pooled estimates of economic status and comorbidity epidemiological characteristics.

				**Random effect model**		
	**Studies**	**Estimate effect**	**95% CI**	**Prevalence (%)**	**95% CI (%)**	**Heterogeneity (I^2^)**
**Comorbidity**						
Depression	3	−0.985	−3.023; 0.086	8.6	1.9; 31.9	99%
Dyskinesias	2	1.59	−0.827; 0.554	55.4	13.5; 90.9	96%
HIV	3	0.445	−1.619; 0.282	28.2	7.1; 66.9	72%
Hypertension	3	−1.344	−3.382; 0.062	6.15	1.3; 24.7	96%
Others	1	3.984	0.623; 0.931	93.1	37.4; 99.7	–
Psychiatric illness	1	−1.061	−4.107; 0.08	8	0.5; 58.3	–
Spinal cord pathology	1	0.723	−2.338; 0.341	34.1	3.1; 89.4	–
Stroke	2	−2.769	−5.115; 0.016	1.6	0.2; 10.3	98%
Essential tremor	3	−2.156	−4.212; 0.028	2.8	0.6; 12.9	98%
Cognitive Impairment	5	−1.381	−2.633; 0.201	20.1	6.7; 46.8	99%
**Economic status**						
Others	9	−2.807	−4.074; 0.057	5.7	1.67; 17.6	99%
Subsistence	27	0.747	−0.717; 0.113	11.3	5.8; 20.9	99%

CI, confidence interval.

## Discussion

This study aimed to provide scientific evidence on regional estimates of NDs prevalence and incidence; investigate factors that contribute to the estimated heterogeneity (study location {Countries}, ND types, study design, sample size, study period); and find evidence of change in NDs prevalence and/or incidence over time concerning gender per age or year. Overall, our findings indicate a higher regional prevalence of NDs with substantial heterogeneity among the included studies. The covariates analysis of study design, size, and period variables significantly account for the pooled prevalence heterogeneity. Also, there is an increasing occurrence of NDs in females per year and age within the region. These findings are consistent with studies elsewhere (67–72). These systematic review and meta-analysis are the first approach to compile epidemiological data on NDs in the East African region. However, accurately quantifying and comparing the burden of NDs across countries could be challenging due to broad, multicultural, and multiethnic regions. Nevertheless, our findings are informative and describe the prevalent cases or occurrences based on retrospective data from studies that have primarily covered regional populations.

Numerous research work on the geographical distribution of NDs have been conducted around the world with little focus on resources and limited regional-based evaluation. Our study gives thorough, current estimates of the prevalence and important epidemiological aspects of NDs in the East African region. The systematic review revealed 42 studies on epidemiology and its prevalence; Tanzania researchers have shown excellent interest in NDs with 31 (73.8%) of the studies, one (2.4%) from Ethiopia, five (11.9%) from Uganda and five (11.9%) from Kenya, while 25 (59.5%) of the studies was meta-analyzed using a random-effect model.

Our analysis reveals a prevalence of 6.09%, of which females were reported to have the highest proportion (61.08%), while the overall random model pool estimates a prevalence of 7.95%, indicating that NDs are becoming a common disease in the region. The prevalence of NDs in the region designates a public health burden, mounting beyond rare diseases based on WHO (73) and European (74) standards. Rare diseases have a substantially lower population incidence than common disorders resulting in higher demands for disease documentation and more extensive reliance on reliable and thorough epidemiological data (75). This may be due to a lack of public awareness and integrated health systems; the few large-scale epidemiological studies that have been conducted to focus on restricted locations, limited populations, and partial epidemiological variables, resulting in inadequate evidence synthesis and outcomes.

An observable upsurge was seen in the yearly status of the disease in 2020, with the female having the highest, confirming the projection report before 2050 (9–11). Also, studies have implicated age (those above the age of 60), gender (female), family, educational level, and rural living as substantial risk factors for developing NDs (8, 76–78). Moreover, evidence (79) predicts that population aging or growth is a driving factor for individuals affected by dementia worldwide. We are not surprised to see females having the highest gender per year/age distribution, as it is consistent with previous reports that suggest sex differences are a potential biological pathway underlying Alzheimer's disease (79–81). Interestingly, our result shows a growing prevalence from age 45, unlike the well-reported cases of NDs burden from age 65 years. It implies that NDs in the region have undergone a shift that may potentially affect life expectancy. NDs could be cellular or molecular and could be caused by a metabolic deficiency or hereditary/genetic or medical issues such as (alcoholism, tumor, stroke), or toxins, chemicals, and viruses which are more common as people get older (5, 82–85). At the same time, many people living in hard-to-reach limited resources settings do not seek medical help early enough. The absence of diagnostic facilities has aggravated the prevalence of NDs-related illnesses, and many cases remain undiagnosed and unmanaged, putting the population at risk of developing into mortality.

Our study reveals NDs prevalence order of (Dementia 52% > Parkinson's 24% > Alzheimer's 16% > Multiple sclerosis 8%), indicating dementia has been the most reported and widespread NDs in the region. This study is in agreement with the report of Lekoubou et al. (13), where dementia was the most common neurological disorder, and Alzheimer's disease accounted for the majority of cases. Also, the incidence of dementia is increasing in middle and north Africa (86). Investigation implicates dementia to have a lifelong course, with risk aggregating around specific periods and frequently accumulation, sometimes decades before clinical symptoms (87). Some risk factors impact early in life, while others occur later due to environmental interaction (42, 88, 89). Again Ethnic differences associated with biological peril factors, including cerebrovascular disease and cognitive impairment, also contribute to dementia and Alzheimer's disease prevalence in the region (8, 77).

Our sensitivity analysis showed substantial heterogeneity among the studies; however, each country and disease type have no significant contribution to the heterogeneity. Furthermore, study design, sample size, and study period accounted for the significant differences. These could explain the broad prevalence range observed in both population and hospital-based research work for NDs, and the heterogeneity could potentially be due to methodology differences. These disparities in NDs frequency are not limited to East Africa, and the same has also been reported in Europe (90). Because most of the studies were community-based prevalence studies and retrospective descriptive studies where the final diagnosis was not based on pathogenesis and variation in the sample size, we have less clarity in the case of Multiple Sclerosis and Huntington's disease, making comparisons and inferences inaccurate.

Due to the enormous socioeconomic expenses that could amplify the increase of NDs and the disease's complexity in the region, it's critical to identify the risk factors that contribute to the related illness development to establish an effective management approach. In several parts of the world, including East Africa, NDs have shown a quick change that could affect life expectancy.

Epidemiological characteristics, comorbidity, and economic status are also linked to the upsurge of NDs in the East African region. Mainly, dyskinesias, HIV, spinal cord pathology, cognitive impairment, chronic choreoathetosis, and abnormalities of sympathetic and parasympathetic functions are the comorbidities linked to NDs in our findings (33, 34, 37, 43, 46, 48, 51, 91). While the majority of the population's livelihood is subsistence farming, which is implicated in the disease progression (25, 38, 45, 49, 55, 60, 66), the increase in NDs prevalence in East Africa could be attributed to the region's changing lifestyles and social factors, including smoking, drinking, pre-existing medical disorders, or environmental factors like air pollution (92–96).

Epidemiological surveys are critical for developing preventative and management strategies for the region's potential epidemic ill health. Due to the current lack of availability of effective NDs-modifying medications, immediate attempts to limit the disease-increasing projection could focus on disease prevention *via* interventions to regulate risk factors (97, 98).

## Strengths and limitations

Our study is the first of its type in East Africa on neurodegenerative diseases. We are confident it will draw the attention of policymakers to intervention strategies in the region. The epidemiological survey emphasizes the region's circumstances and highlights research priorities. Conversely, we examined a regionally-based population study by including community diverse data sources to suggest clear information about the present burden and risk factors to stakeholders for future preparedness.

The Egger test signifies a publication bias for the occurrence proportion and period prevalence, and publication bias implies possible unpublished research with different findings. Again, combining research from different countries and assessing changes over time is also tricky because of the lack of uniform or specific information. Here, some studies did not provide the total population or cases, specific age and sex, and studies period, and were excluded from the prevalence estimates. Also, our analysis covered only studies published in English. However, two reviewers' thorough assessment of the study's quality and independent data extraction were among the study's strengths.

## Conclusion

The findings reveal a possible regional increase in NDs as an implication of common disorders in the region, with significant gaps in the epidemiological drivers. Particularly the occurrence of NDs in low-income sittings requires advances in screening tools and public health efforts in intervention strategies. We recommend to policymakers make provisions for quality healthcare in rural settings to improve early diagnosis and care for the affected individuals.

## Data availability statement

The original contributions presented in the study are included in the article/Supplementary files, further inquiries can be directed to the corresponding author/s.

## Author contributions

HO conceived, designed the study, and drafted the manuscript. HO, AA, and SI conducted the dataset searches. HO and AA extracted and conducted data analysis. All authors read, revised, and approved the manuscript.

## Conflict of interest

The authors declare that the research was conducted in the absence of any commercial or financial relationships that could be construed as a potential conflict of interest.

## Publisher's note

All claims expressed in this article are solely those of the authors and do not necessarily represent those of their affiliated organizations, or those of the publisher, the editors and the reviewers. Any product that may be evaluated in this article, or claim that may be made by its manufacturer, is not guaranteed or endorsed by the publisher.

## Abbreviations

ND: Neurodegenerative Disease
AD: Alzheimer's disease
PD: Parkinson's disease
HD: Huntington's disease
ALS: Amyotrophic Lateral Sclerosis
US: United States
TTH: Tension-Type Headache
SCI: spinal cord injuries
US: United States
WOS: Web of Science
WHO: World Health Organization.
