# Supplementary material for: Epidemiology of neurodegenerative diseases in the East African region: A meta-analysis
Source: Front Neurol. 2022 Nov 17;13:1024004. doi: 10.3389/fneur.2022.1024004 (PMC9718573; doi:10.3389/fneur.2022.1024004)
Supplement: Supplementary file 1 [file Data_Sheet_1.PDF]

**Epidemiology of neurodegenerative diseases in East African region: meta-analysis**

Supplementary files

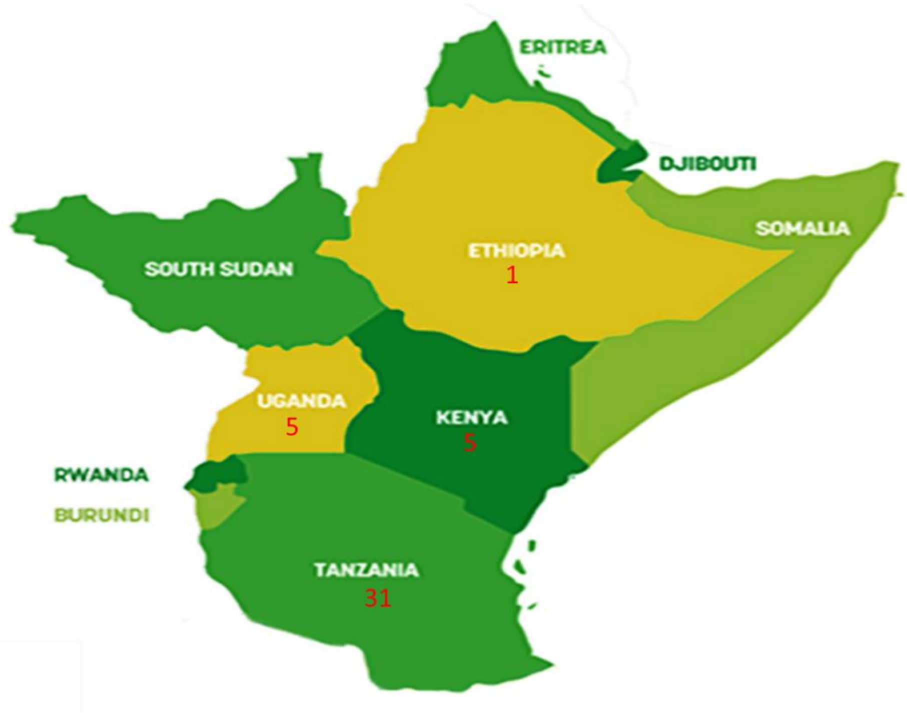


S Figure 1. Selected studies hotspot in the East Africa region on the prevalence of NDs.

S Table 1. The Newcastle-Ottawa Scale (NOS) quality assessment of the included studies.

| **First author year** | **Selection** | | | | | | **Comparability** | **Outcome** | |
|---|---|---|---|---|---|---|---|---|---|
| **Authors** | **Nation/Region** | **Study type** | **Degenerative Disease** | **Study Period** | **Sample size** | **Case** | **Comparability of cohorts** | **Prevalence /incident** | **Score** |
| Community-based prevalence study | | | | | | | | | |
| Kioy *et al.*, 2001 | * | * | * | * | * | * | NA | * | 7 |
| JAMAL *et al.*, 2021 | * | * | * | * | * | * | NA | * | 7 |
| Dotchin *et al.*, 2014 | * | * | * | NA | * | * | NA | * | 6 |
| Dotchin *et al.*, 2014 | * | * | * | NA | * | * | NA | * | 6 |
| Scrimgeour *et al.*, 1981 | * | * | * | NA | NA | * | NA | NA | 5 |
| Aris *etal.*, 2013 | * | * | * | * | * | * | NA | * | 7 |
| Winkler *et al.*, 2009 | * | * | * | * | * | * | NA | * | 6 |
| Miller *et al.*, 2012 | * | * | * | NA | NA | * | NA | NA | 4 |
| Longdon *et al.*, 2012 | * | * | * | NA | * | * | NA | * | 6 |
| Paddick *et al.*, 2020 | * | * | * | * | * | * | NA | * | 7 |
| Cross-sectional Population-Based Study | | | | | | | | | |
| Mubangizi *et al.*, 2020 | * | * | * | NA | * | * | NA | * | 6 |
| Cross-sectional qualitative study | | | | | | | | | |
| Mushi *et al.*, 2014 | * | * | * | * | * | * | NA | * | 7 |
| Kaddumukasa *et al.*, 2016 | * | * | * | * | * | * | NA | * | 7 |
| Kankongi *et al.*, 2020 | * | * | * | * | * | * | NA | * | 7 |
| Hospital-based study | | | | | | | | | |
| Paddick *et al.*, 2015 | * | * | * | * | * | * | NA | * | 7 |
| Winkler *et al.*, 2009 | * | * | * | * | * | * | NA | * | 7 |
| Sacktor *et al.*, 2014 | * | * | * | * | * | * | NA | * | 7 |
| Dotchin *et al.*, 2007 | * | * | * | NA | NA | * | NA | NA | 4 |
| Questionnaire | | | | | | | | | |
| Chen *et al.*, 2010 | * | * | * | NA | * | * | NA | * | 6 |
| Dotchin *et al.*, 2008 | * | * | * | * | * | * | NA | * | 7 |
| Mashana *et al.*, 2011 | * | * | * | * | * | * | NA | * | 7 |
| Sacktor *et al.*, 2009 | * | * | * | * | * | * | NA | * | 7 |

| | | | | | | | | | |
|---|---|---|---|---|---|---|---|---|---|
| Hindley *et al*., 2016 | * | * | * | * | * | * | NA | * | 7 |
| Interventional study | | | | | | | | | |
| Paddick *et al*., 2017 | * | * | * | NA | * | * | NA | * | 6 |
| Observational repeated measure design | | | | | | | | | |
| Rochester *et al*., 2010 | * | * | * | * | * | * | NA | * | 7 |
| Paddick *et al*., 2015 | * | * | * | * | * | * | NA | * | 7 |
| Screening based on app technology | | | | | | | | | |
| Paddick *et al*., 2020 | * | * | * | * | * | * | NA | * | 7 |
| Cohort | | | | | | | | | |
| Fothergill *et al*., 2021 | * | * | * | * | NA | * | NA | NA | 5 |
| Kisoli *et al*., 2015 | * | * | * | * | * | * | NA | * | 7 |
| kisoli *et al*., 2015 | * | * | * | * | * | * | NA | * | 7 |
| Dotchin *et al*., 2011 | * | * | * | * | * | * | NA | * | 7 |
| Kellet-Wright *et al*., 2020 | * | * | * | * | * | * | NA | * | 7 |
| Paddick *et al*., 2014 | * | * | * | NA | * | * | NA | * | 6 |
| Paddick *et al*., 2014 | * | * | * | NA | * | * | NA | * | 6 |
| Paddick *et al*., 2014 | * | * | * | * | * | * | NA | * | 7 |
| Paddick *et al*., 2014 | * | * | * | * | * | * | NA | * | 7 |
| Paddick *et al*., 2014 | * | * | * | * | * | * | NA | * | 7 |
| Paddick *et al*., 2017 | * | * | * | * | * | * | NA | * | 7 |
| Paddick *et al*., 2018 | * | * | * | NA | * | * | NA | * | 6 |
| Masika *et al*., 2020 | * | * | * | * | * | * | NA | * | 7 |
| Kwasa *et al*., 2012 | * | * | * | * | * | * | NA | * | 7 |
| Matuja *et al*., 2008 | * | * | * | * | * | * | NA | * | 7 |

NA = Non available

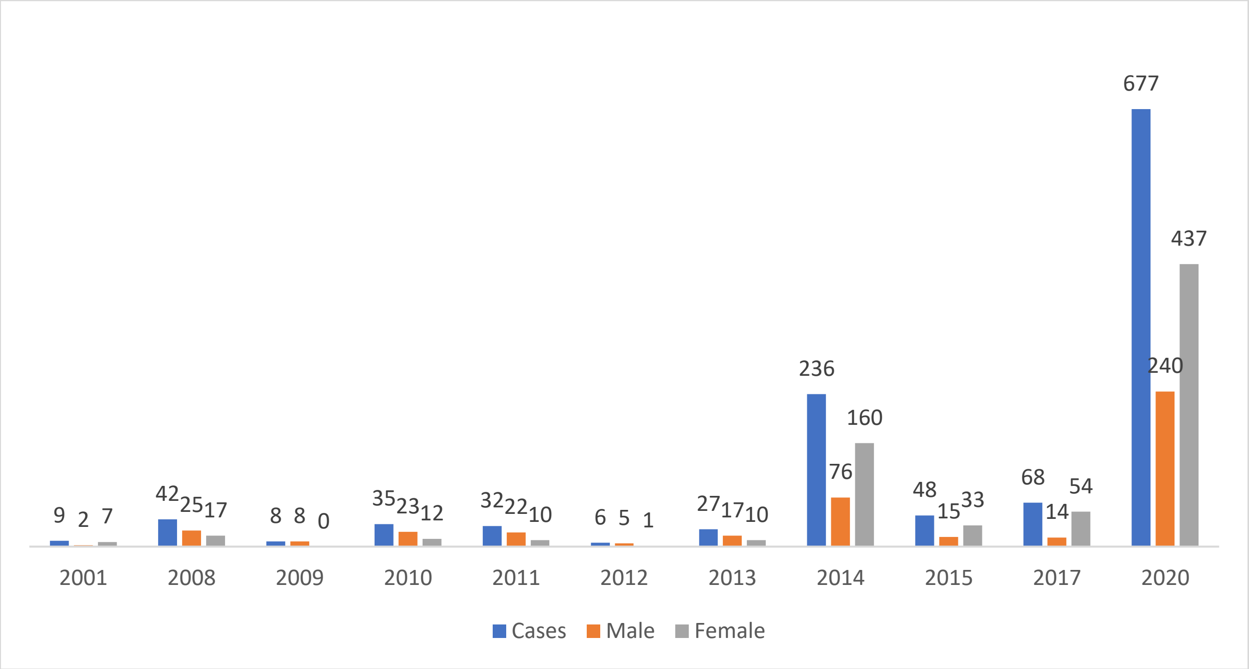


S Figure 2. Gender per year distribution of NDs in East Africa regional meta-analysis pool estimate.

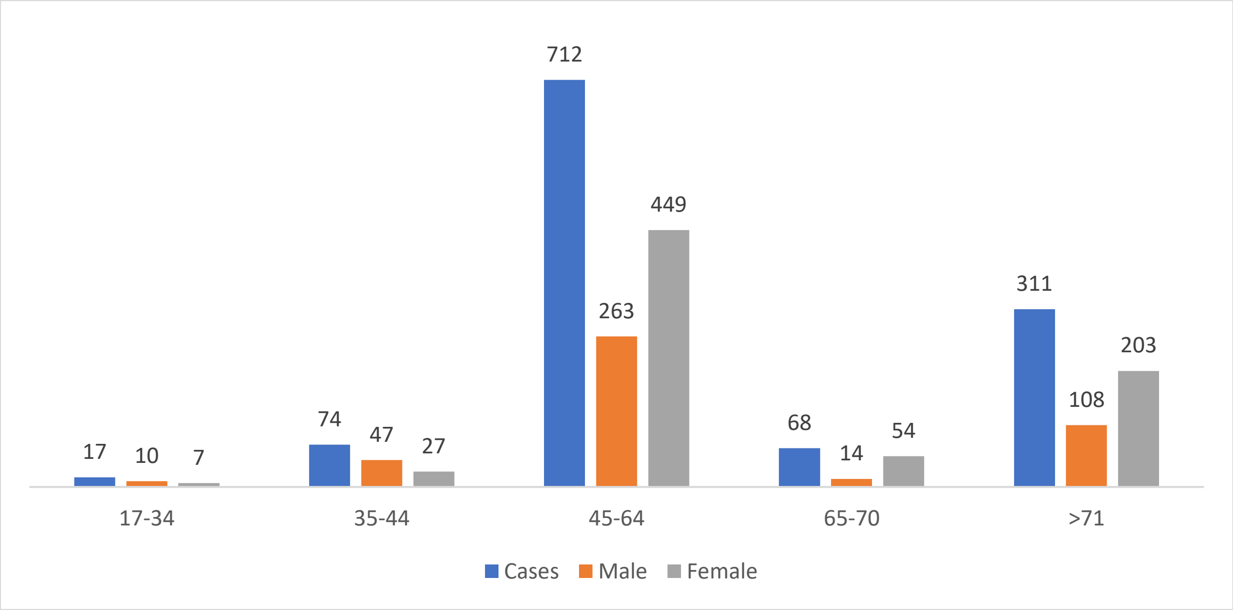


S Figure 3. Gender per age distribution of NDs in East Africa regional meta-analysis pool estimate.

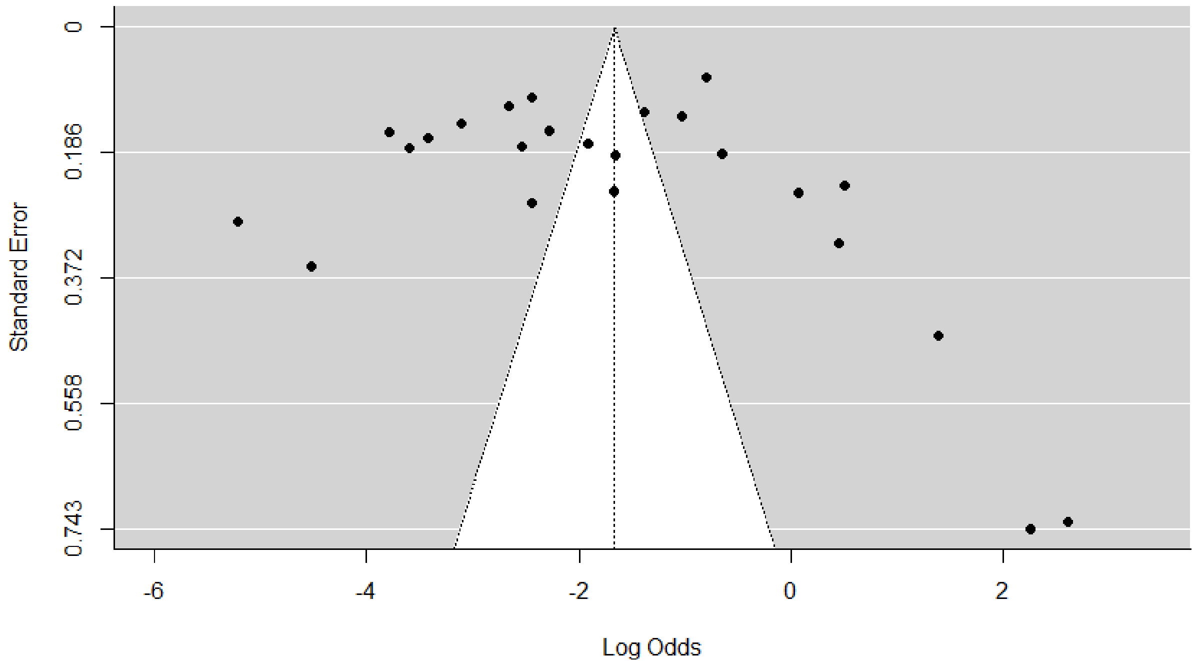


S Figure 4. Publication bias testing for East Africa regional base NDs prevalence studies.

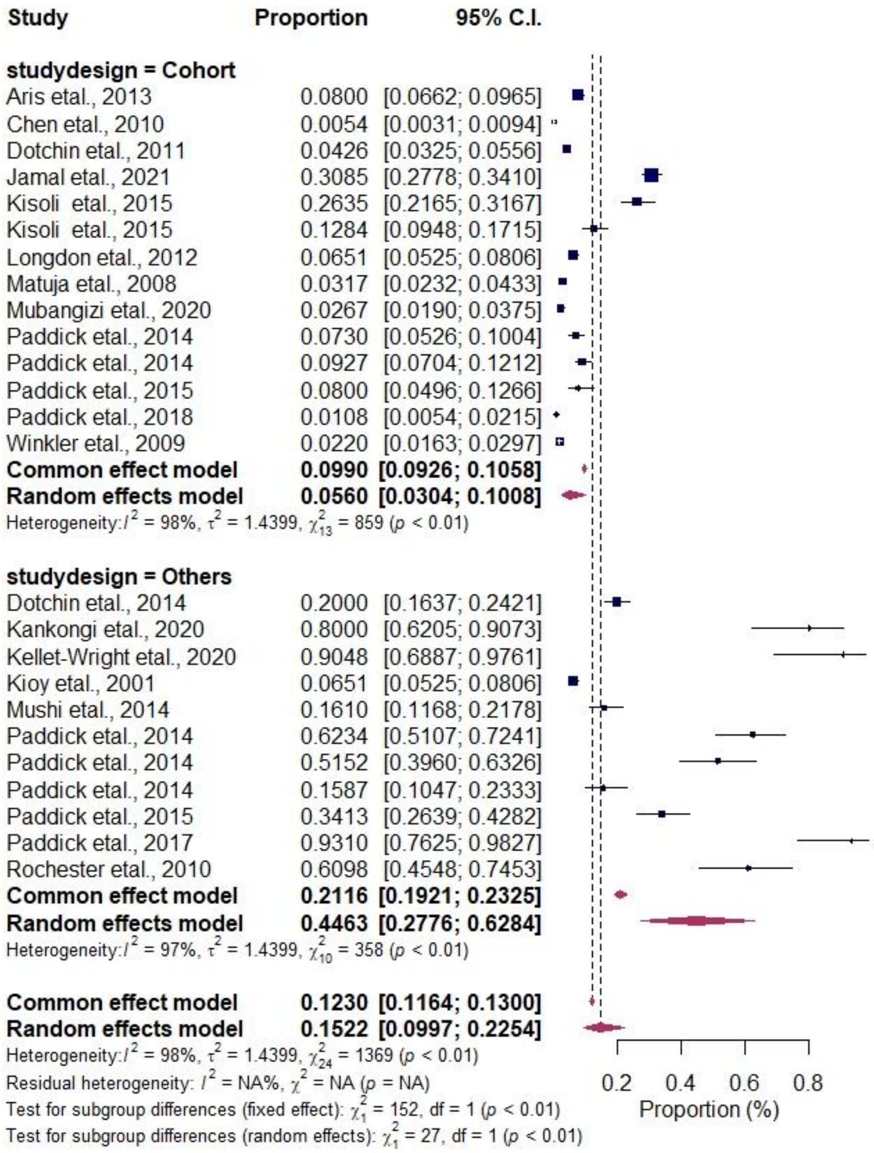


S Figure 5. Forest plot of the subgroup analysis by study design

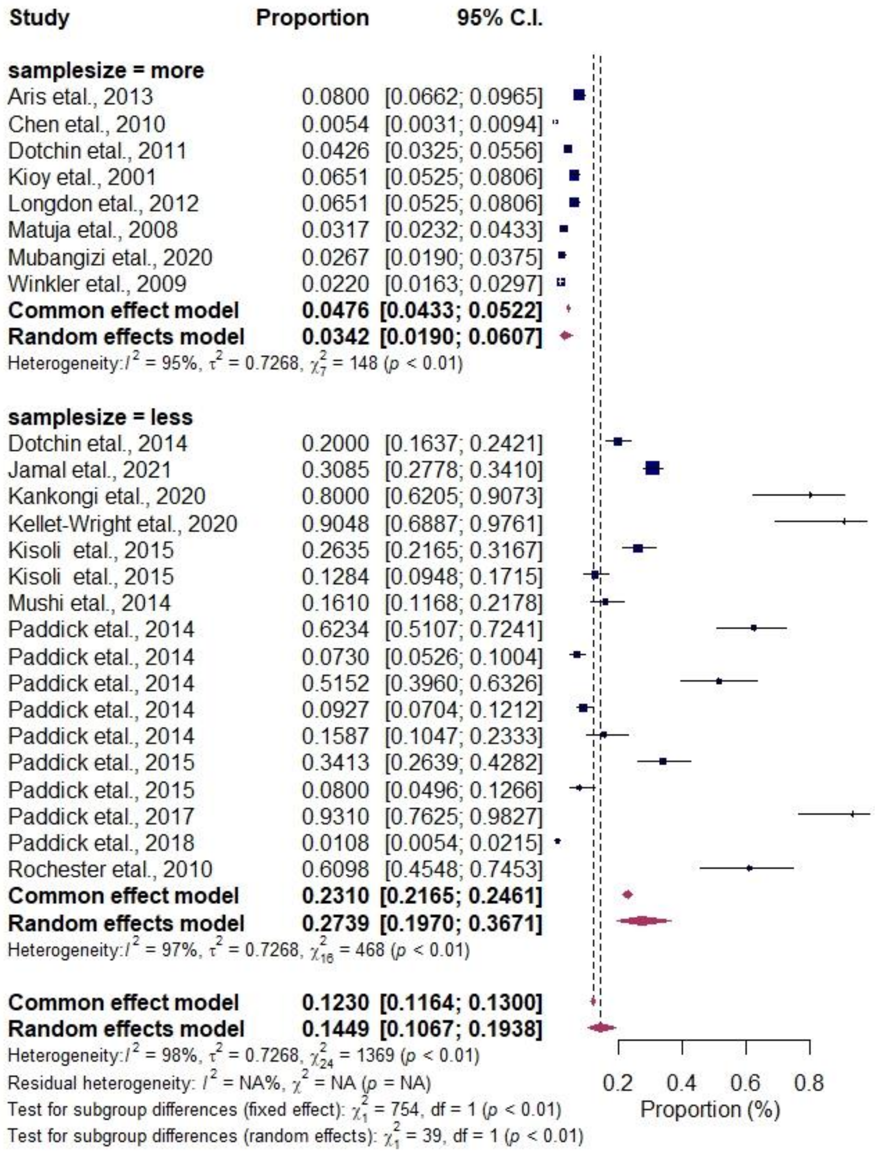


S Figure 6. Forest plot of the subgroup analysis by sample size

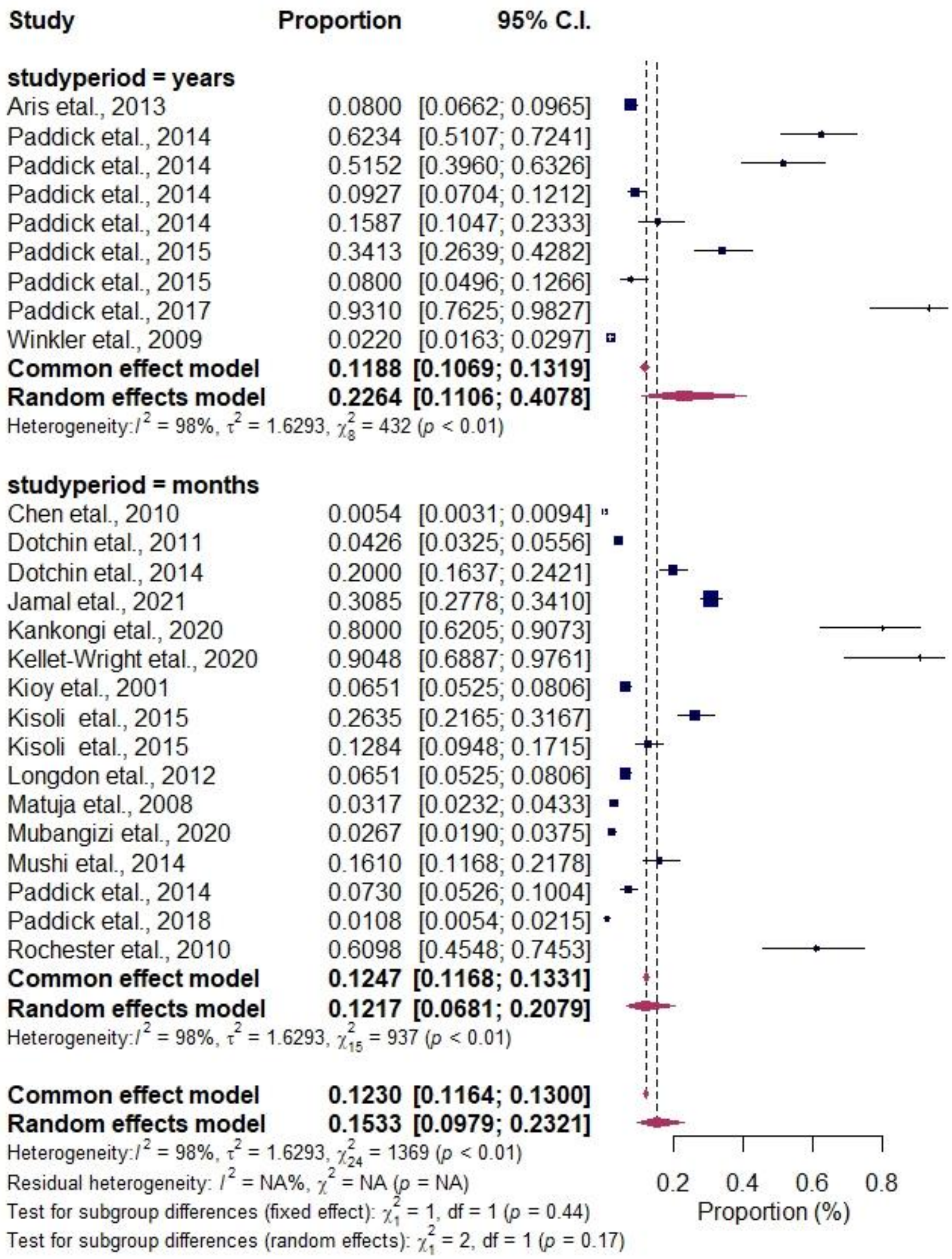


S Figure 7. Forest plot of the subgroup analysis by study period

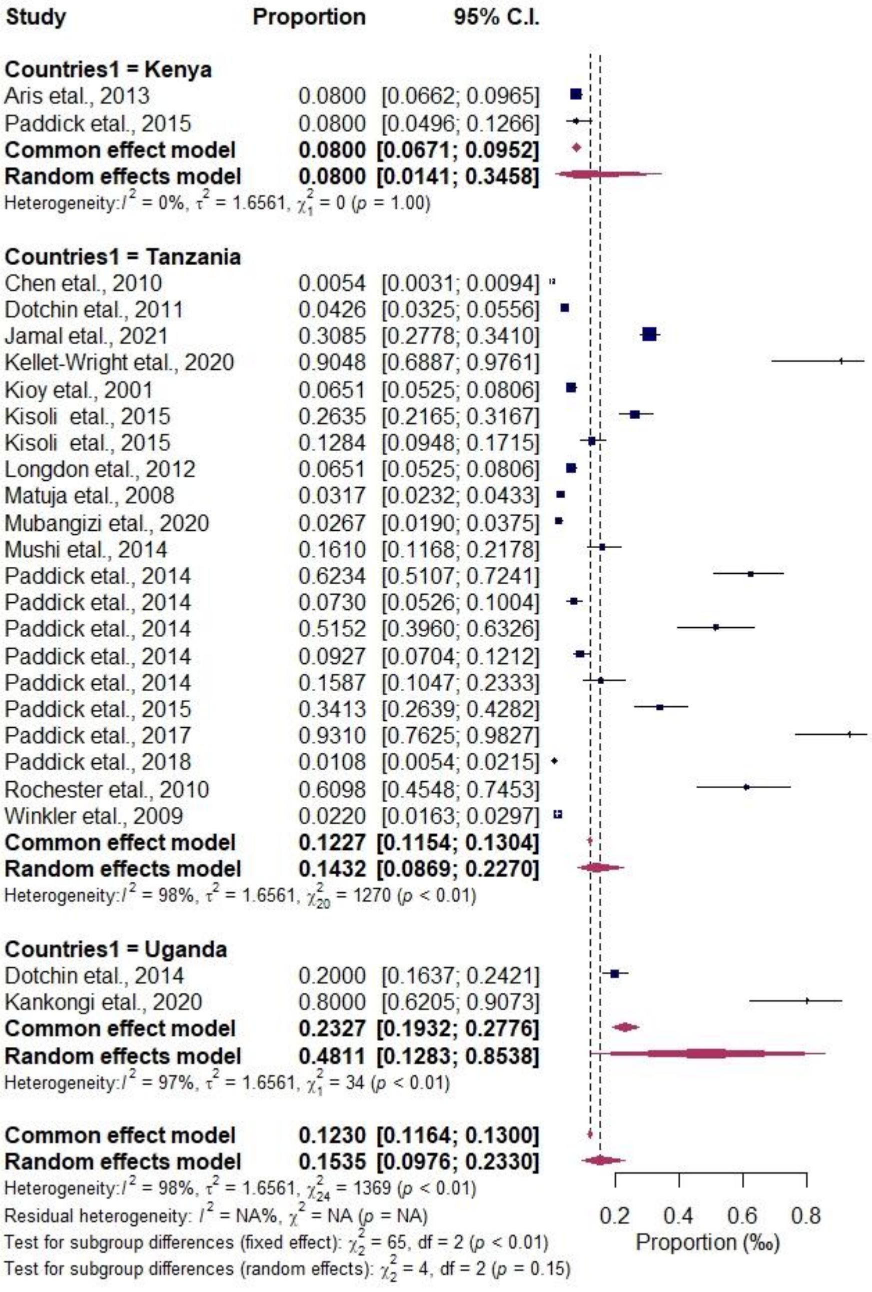


S Figure 8. Forest plot of the subgroup analysis by East Africa region
